# *Xanthomonas* *oryzae* pv. *oryzae* Type III Effector XopN Targets OsVOZ2 and a Putative Thiamine Synthase as a Virulence Factor in Rice

**DOI:** 10.1371/journal.pone.0073346

**Published:** 2013-09-03

**Authors:** Hoon Cheong, Chi-Yeol Kim, Jong-Seong Jeon, Byoung-Moo Lee, Jae Sun Moon, Ingyu Hwang

**Affiliations:** 1 Department of Agricultural Biotechnology, Seoul National University, Seoul, Korea; 2 Graduate School of Biotechnology & Crop Biotech Institute, Kyung Hee University, Yongin, Korea; 3 National Academy of Agricultural Science, Rural Development Administration, Suwon, Korea; 4 Plant Systems Engineering Research Center, Korea Research Institute of Bioscience and Biotechnology, Daejeon, Korea; Indian Institute of Science, India

## Abstract

*Xanthomonas* *oryzae* pv. *oryzae* (Xoo) is spread systemically through the xylem tissue and causes bacterial blight in rice. We evaluated the roles of *Xanthomonas* outer proteins (Xop) in the *Xoo* strain KXO85 in a Japonica-type rice cultivar, Dongjin. Five *xop* gene knockout mutants (*xopQ*_*KXO85*_, *xopX*_*KXO85*_, *xopP1*_*KXO85*_, *xopP2*_*KXO85*_, and *xopN*_*KXO85*_) were generated by EZ-Tn*5* mutagenesis, and their virulence was assessed in 3-month-old rice leaves. Among these mutants, the *xopN*_*KXO85*_ mutant appeared to be less virulent than the wild-type KXO85; however, the difference was not statistically significant. In contrast, the *xopN*_*KXO85*_ mutant exhibited significantly less virulence in flag leaves after flowering than the wild-type KXO85. These observations indicate that the roles of Xop in *Xoo* virulence are dependent on leaf stage. We chose the *xopN* gene for further characterization because the *xopN*_*KXO85*_ mutant showed the greatest influence on virulence. We confirmed that XopN_KXO85_ is translocated into rice cells, and its gene expression is positively regulated by HrpX. Two rice proteins, OsVOZ2 and a putative thiamine synthase (OsXNP), were identified as targets of XopN_KXO85_ by yeast two-hybrid screening. Interactions between XopN_KXO85_ and OsVOZ2 and OsXNP were further confirmed *in planta* by bimolecular fluorescence complementation and *in vivo* pull-down assays. To investigate the roles of OsVOZ2 in interactions between rice and *Xoo*, we evaluated the virulence of the wild-type KXO85 and *xopN*_*KXO85*_ mutant in the *OsVOZ2* mutant line PFG_3A-07565 of Dongjin. The wild-type KXO85 and *xopN*_*KXO85*_ mutant were significantly less virulent in the mutant rice line. These results indicate that XopN_KXO85_ and OsVOZ2 play important roles both individually and together for *Xoo* virulence in rice.

## Introduction

*Xanthomonas* *oryzae* pv. *oryzae* (Xoo) causes bacterial leaf blight, which is one of the most serious diseases in rice (*Oryza sativa* L.). This bacterium invades the xylem of rice leaves through hydathodes or wounds. The strain of *Xoo* KXO85 (KACC10331) was isolated from diseased rice leaves in Korea, and its whole genome sequence was published in 2005 [1].

Plant pathogenic bacteria belonging to the genera *Pseudomonas* , *Xanthomonas* , *Erwinia* , and *Ralstonia* possess the type III protein secretion system (T3SS) that is critical for full virulence and bacterial colonization in their host plants [2–6]. The T3SS of plant pathogenic species of *Pseudomonas* , *Xanthomonas* , *Erwinia* , and *Ralstonia* is highly conserved and involved in translocation of T3SS-dependent effector proteins from bacterial cells into plant cells [7–12]. These effector proteins are categorized into two groups: transcription activator-like (TAL) effectors and non-TAL effectors [11,13–15]. In *Xoo*, T3SS that is essential for virulence is encoded by hypersensitive response and pathogenicity (hrp) genes, the expression of which is controlled by HrpX [13,16,17].

T3SS-dependent plant bacterial effectors are important for bacterial growth, colonization, virulence, and race specificity in their host plants [18–23]. However, the biochemical functions of most T3SS-dependent plant bacterial effectors in their hosts have not been well characterized. *Xanthomonas* outer proteins (Xop) are known as non-TAL bacterial effector proteins that are delivered to the plant cell via Hrp T3SS. The major roles of non-TAL bacterial effectors involve modulation of signaling in the plant defense response [11,24]. For example, XopX_Xcv_ from *X. campestris* pv. *vesicatoria* (Xcv) affects the virulence of *Xcv* on pepper (*Capsicum annuum*) and tomato ( *Lycopersicum* *esculentum* ) and targets basic innate immunity in plants [24]. XopD_Xcv_ is a small ubiquitin-like modifier (SUMO) protease in *Xcv* that promotes bacterial growth in tomato and slows leaf chlorosis and necrosis in tomato at late stages of infection [25]. Another T3SS-dependent non-TAL effector, XopN_Xcc_, plays important roles in colonization and virulence of *X. campestris* pv. *campestris* (Xcc) in their hosts [26]. XopN is highly conserved among *Xanthomonas* species [27]. In addition, XopN_Xcv_ may suppress pathogen-associated molecular pattern (PAMP)-triggered immunity in tomato [28].

Compared to known host targets of TAL effectors in xanthomonads, there have been few studies on non-TAL effector targets in xanthomonads. XopD_Xcv_ may target nuclear SUMOylated proteins [25]. In *Xcc* and *Arabidopsis* interactions, XopD_XccB100_ targets the transcription factor MYB30 to suppress host defense [29]. Recently, it was found that tomato transcription factor SlERF4 was identified as a target of XopD_Xcv_ in tomato [30]. Non-TAL effector Xoo1488 of *Xoo* MAFF311018 targets two receptor-like cytoplasmic kinases (RLCKs), Os01g0936100 (OsRLCK55) and Os05g0372100 (OsRLCK185), to inhibit OsRLCK185 phosphorylation and the downstream MAPK signaling [31]. Other reported host targets of XopN_Xcv_ are tomato atypical receptor-like kinase (TARK1) and four 14-3-3 isoforms (TFT1, TFT3, TFT5, and TFT6) [28].

In *Xoo*, considerable efforts have been made to characterize functional roles of TAL effectors in various strains [32,33]. The contribution of each TAL effector protein to *Xoo* virulence varies; some are critical for virulence, while others have relatively moderate roles [32,34]. However, the roles of non-TAL effectors in *Xoo* virulence have been poorly investigated. When 18 non-TAL effectors were evaluated for virulence in the Philippine strain PXO99^A^, deletion of both copies of *xopZ*_*PXO99*_ conferred significant reduction of virulence, whereas the other non-TAL effectors showed little influence on virulence in 4-week-old rice leaves [35]. Disease severity of *Xoo* in susceptible cultivars varies depending on leaf stage [36–39]. This led us to assess the virulence of each *xop* mutant at the adult stage in the field with the expectation of more distinct and different disease response outcomes compared to virulence assay results at the young leaf stage. Here, we report the contribution of XopN_KXO85_ to *Xoo* virulence in the Korean strain KXO85 at flag leaf stage in the field, identification of targets of XopN_KXO85_ in rice, and their important roles for *Xoo* virulence.

## Results

### Mutagenesis of five *xop* genes in the Korean *Xoo* strain KXO85

**Figure 1:**
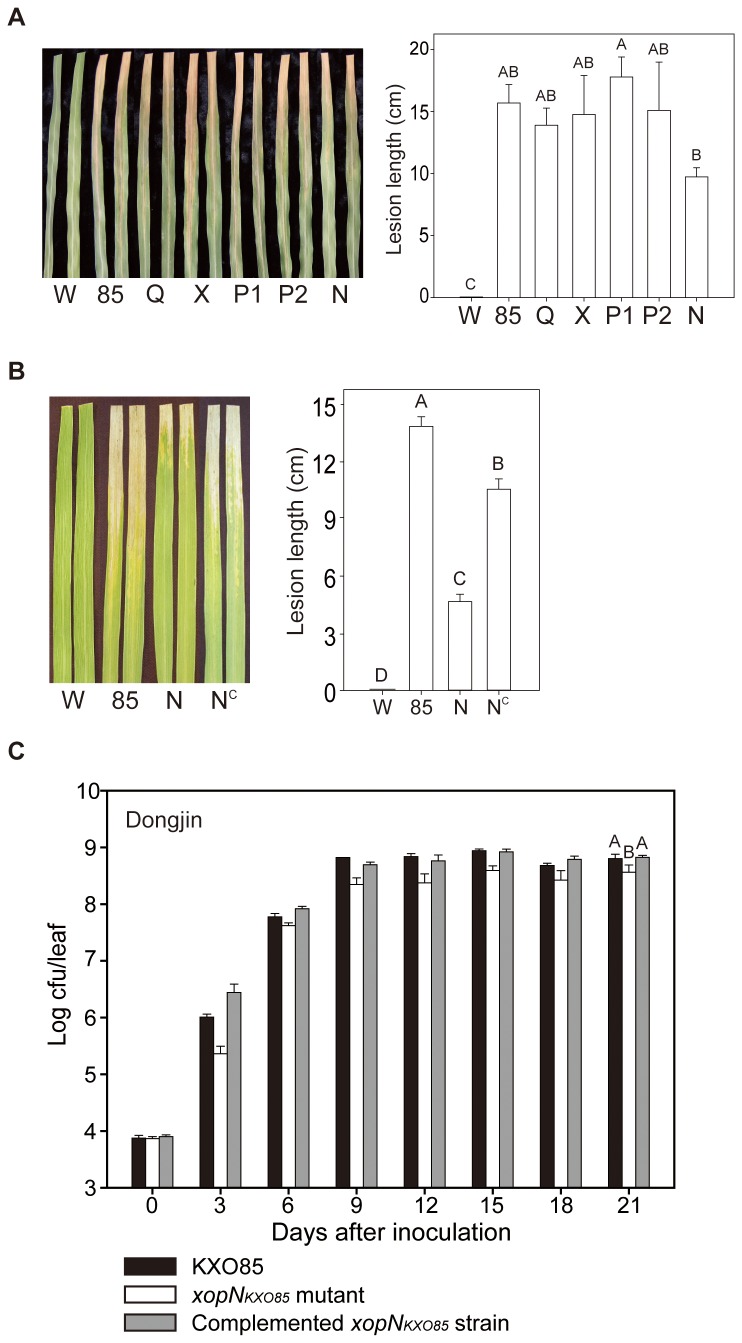
Pathogenicity test for *xop* mutants of *Xoo* KXO85 in rice. **A**. Disease severity of each *xop* mutant in 3-month-old rice leaves. W, water; 85, wild-type KXO85; Q, KXO85 *xopQ*_*KXO85*_::EZ-Tn*5*; X, KXO85 *xopX*_*KXO85*_::EZ-Tn*5*; P1, KXO85 *xopP1*_*KXO85*_::EZ-Tn*5*; P2, KXO85 *xopP2*_*KXO85*_::EZ-Tn*5*; N, KXO85 *xopN*_*KXO85*_::EZ-Tn*5*. **B**. Disease severity of the *xopN*_*KXO85*_ mutants in the flag leaves of rice grown in a paddy field. W, water; 85, KXO85; N, KXO85 *xopN*_*KXO85*_::EZ-Tn*5*; and N^C^, KXO85 *xopN*_*KXO85*_::EZ-Tn*5* (pML122G2). Photographs were taken and lesion lengths were determined 21 days after inoculation. Vertical error bars indicate the standard deviations (SD). The data are the averages of 12–15 replicates for each treatment. Columns and lines not connected by the same letter are significantly different (P<0.05) as determined by a one-way ANOVA (P<0.001) followed by post hoc Tukey HSD analysis. **C**. Bacterial growth patterns of the KXO85, *xopN*_*KXO85*_ mutant, and complemented *xopN*_*KXO85*_ mutant strains in flag leaves of wild-type Dongjin. The data are shown as the average values for three replicates; vertical bars indicate the error ranges (±SD). The bacterial populations were assessed every 3 days after inoculation. Different letters at day 21 indicate significant differences (P<0.05) as determined by a one-way ANOVA (P<0.001) followed by post hoc Tukey HSD analysis.

Five *xop* genes, *xopQ*_*KXO85*_ (XOO4466), *xopX*_*KXO85*_ (XOO4287), *xopP1*_*KXO85*_ (XOO3425), *xopP2*_*KXO85*_ (XOO3426), and *xopN*_*KXO85*_ (XOO0343) (Table S1), were characterized among 18 *xop* homologs in the strain KXO85 (www.xanthomonas.org/t3e.html), which showed significant homology with reported *xop* genes. EZ-Tn5 insertion mutants of *xopQ*_*KXO85*_, *xopX*_*KXO85*_, *xopP1*_*KXO85*_, *xopP2*_*KXO85*_, and *xopN*_*KXO85*_ (Figure S1) were generated in the strain KXO85, and then the virulence of each *xop* gene knockout mutant was evaluated in 3-month-old leaves of the Japonica-type rice cultivar Dongjin. Mutations in the *xopQ*_*KXO85*_, *xopX*_*KXO85*_, *xopP1*_*KXO85*_, *xopP2*_*KXO85*_, or *xopN*_*KXO85*_ gene did not significantly affect virulence (Figure 1A). When the *xopN*_*KXO85*_ mutant was inoculated into the flag leaves of Dongjin in the field, the mutant was significantly less virulent than the wild-type KXO85 (Figure 1B). Virulence the *xopN*_*KXO85*_ mutant carrying each wild-type *xop* gene in a multicopy plasmid was recovered to the wild-type level (Figure 1B). These observations indicate that *xopN*_*KXO85*_ exhibits important roles for virulence of *Xoo*. Therefore, we chose *xopN*_*KXO85*_ for further characterization. The bacterial population of the *xopN*_*KXO85*_ mutant was reduced up to 21 days after inoculation of flag leaves compared to the growth of wild-type strain KXO85 in Dongjin (Figure 1C).

### Expression of *xopN_KXO85_* is regulated by HrpX_KXO85_

As expression of *hrp* and *xop* genes in *Xcv* and other xanthomonads is controlled by two regulatory genes, *hrpG* and *hrpX*, we examined whether *xopN*_*KXO85*_ is regulated by HrpX_KXO85_ in *Xoo* KXO85. Expression of *xopN*_*KXO85*_ was below the limit of detection as assessed by quantitative real-time polymerase chain reactions (PCR) in the wild-type KXO85 or in the *hrpX*_*KXO85*_ mutant strain in rich PSB medium (Figure S2A). In the *hrp*-inducing medium XOM2, *xopN*_*KXO85*_ expression in the wild-type KXO85 was approximately 3-fold higher than that in the *hrpX*_*KXO85*_ mutant (Figure S2A). We found a conserved *cis*-regulatory element plant-inducible promoter (PIP) box (TTCGG-N_15_-TTCTG) in the region from -263 to -239 upstream of the start codon of *xopN*_*KXO85*_ (Figure S2B). These results indicate that *xopN*_*KXO85*_ belongs to the HrpX_KXO85_ regulon in *Xoo* KXO85.

### XopN_KXO85_ is a T3SS-dependent effector translocated into plant cells in the strain KXO85

To investigate whether XopN_KXO85_ is translocated into plant cells in a T3SS-dependent manner, we conducted a XopN_KXO85_ translocation assay using the XopN-Cya fusion protein in the wild-type strain KXO85 and the T3SS-deficient mutant KXO85 *hrpB5*_*KXO85*_::EZ-Tn*5* in rice (Figure S3A). The level of cAMP increased in the wild-type strain KXO85, whereas no change in cAMP level was detected in the T3SS-deficient mutant KXO85 *hrpB5*_*KXO85*_::EZ-Tn*5* (Figure S3B). This indicates that XopN_KXO85_ is translocated into rice cells in a T3SS-dependent manner.

### Identification of XopN_KXO85_ targets in rice by yeast two-hybrid screening

**Figure 2:**
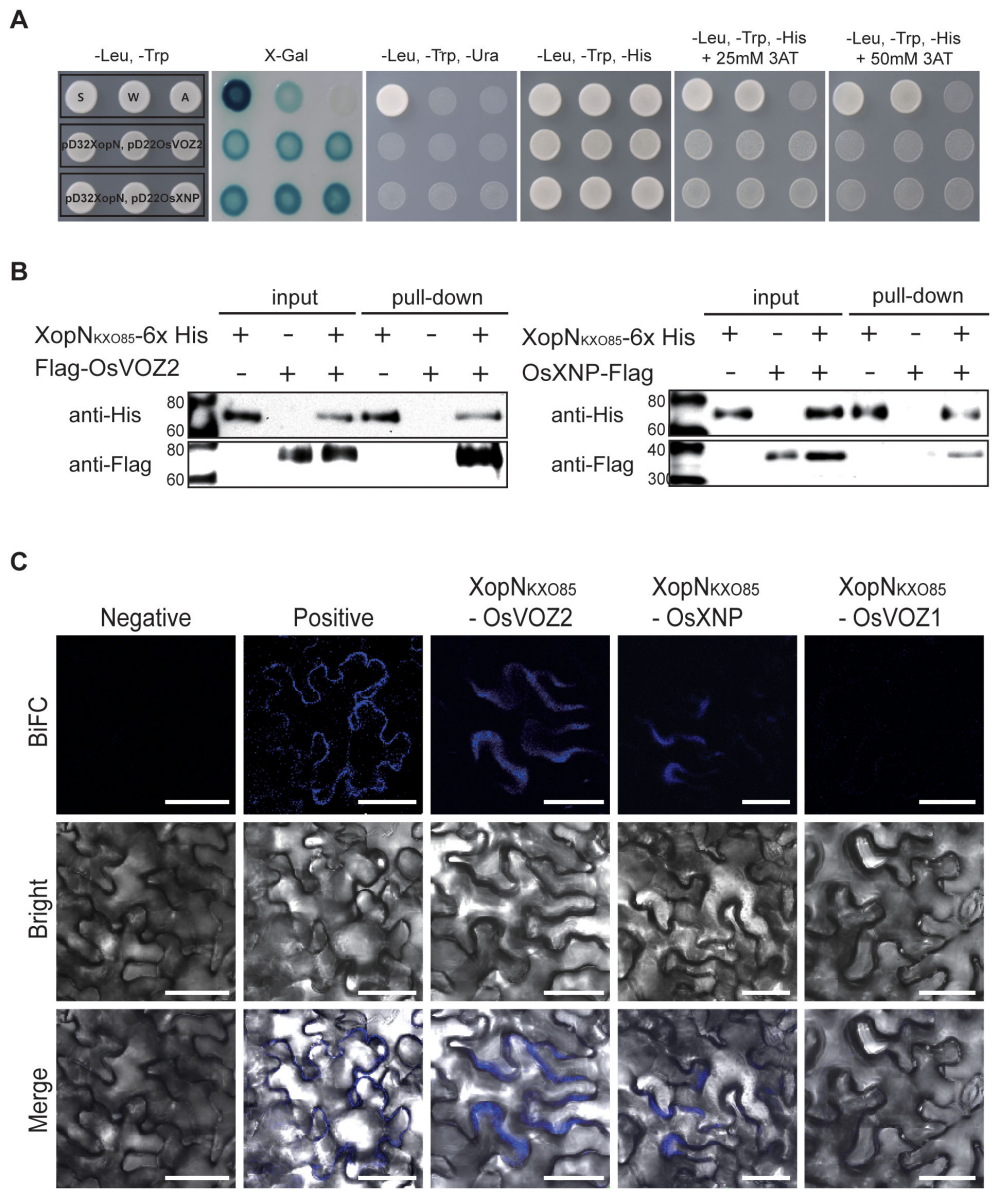
Interactions between XopN_KXO85_ and OsVOZ2 and OsXNP. **A**. Screening for interactors of XopN_KXO85_ in rice using a yeast two-hybrid system. S (strong: pEXP ^TM^32/Krev1 + pEXP ^TM^22/RalGDS-wt), W (weak: pEXP ^TM^32/Krev1 + pEXP ^TM^22/RalGDS-m1), and A (absent: pEXP ^TM^32/Krev1 + pEXP ^TM^22/RalGDS-m2) indicate the strength of each interaction. Three independent and representative colonies are shown for each bait–prey combination. **B**. *In vivo* pull-down analysis of XopN_KXO85_ and OsVOZ2 (left panel) and XopN_KXO85_ and OsXNP (right panel). Total proteins from *N* *. benthamiana* leaves co-expressing XopN_KXO85_-6× His and Flag-OsVOZ2 or XopN_KXO85_-6× His and OsXNP-Flag protein were purified by Ni^+^ affinity chromatography followed by Western blotting using anti-His and anti-Flag antibodies. The expected molecular weights were as follows: XopN_KXO85_-6× His = 78.7 kDa; Flag-OsVOZ2 = 74.6 kDa; OsXNP-Flag = 40.1 kDa; +, protein expressed; and -, vector control. **C**. BiFC analysis of XopN_KXO85_ -OsVOZ2, XopN_KXO85_ -OsXNP, and XopN_KXO85_ -OsVOZ1 interactions in *N* *. benthamiana* leaves. Negative, pDEST-SCYNE(R)^GW^ + pDEST-SCYCE(R)^GW^; positive, pEXP-SCYNE(R)-Cnx7 + pEXP-SCYCE(R)-Cnx6. Bars = 50 µm.

To identify XopN_KXO85_ target proteins in rice, we carried out yeast two-hybrid screening using GAL4-XopN as a bait protein and a rice cDNA library constructed in the prey vector in the *Saccharomyces cerevisiae* strain MaV203. We found two possible candidates: *Oryza sativa* vascular plant one zinc finger protein 2 (OsVOZ2: NP_001056041, Os05g0515700) and *O. sativa* XopN_KXO85_ binding protein (OsXNP: NP_001059841, Os07g0529600) (Figure 2A and Table S2). The *OsVOZ2* gene is 3,630 bp in length consisting of four exons and three introns and encodes a protein of 69,901 Da. OsVOZ2 is a homolog of *Arabidopsis thaliana* vascular plant one zinc finger protein 2 (AtVOZ2; At2g42400) that has a conserved zinc finger domain (Figure S5 and Figure S6). The *OsXNP* gene is 1,489 bp in length with two exons and one intron and possibly encodes a putative protein of 37,224 Da that has significant homology with thiamine biosynthetic enzyme in *Saccharum* hybrid cultivar GT28 (Table S2). XopN_KXO85_, OsVOZ2, and OsXNP were expressed in yeast as confirmed by immunoblot using anti-GAL4BD and anti-GAL4AD antibodies (Figure S4).

### XopN_KXO85_ physically interacts with two rice proteins OsVOZ2 and OsXNP *in planta*

To confirm the specific interactions between XopN_KXO85_ and OsVOZ2 and XopN_KXO85_ and OsXNP *in planta*, we performed affinity pull-down experiments in *Nicotiana* *benthamiana* ( *N* *. benthamiana* ) leaves. Cells of *Agrobacterium tumefaciens* strain C58C1 (pCH32) carrying pGWB8-XopN (*xopN*_*KXO85*_-6× His in pGWB8) or pGWB12-OsVOZ2 (Flag-*OsVOZ2* in pGWB12) were co-infiltrated into *N* *. benthamiana* leaves. For pull-down experiments to investigate interactions between XopN_KXO85_ and OsXNP, *A. tumefaciens* cells harboring pGWB8-XopN and pGWB11-OsXNP (*OsXNP*-Flag in pGWB11) were co-infiltrated into *N* *. benthamiana* leaves. Eluted soluble proteins bound to Ni-nitrilotriacetic acid (Ni-NTA) superflow agarose slurry were subjected to immunoblotting analysis using anti-His or anti-Flag antibodies. Both Flag-OsVOZ2 and OsXNP-Flag proteins were pulled down by XopN_KXO85_-6xHis (Figure 2B). These results indicate that XopN_KXO85_ physically interacts with OsVOZ2 or OsXNP in *N* *. benthamiana* leaves.

### Visualization of the interactions of OsVOZ2 and OsXNP with XopN_KXO85_

A bimolecular fluorescence complementation (BiFC) assay was performed to examine the interactions between XopN_KXO85_ and OsVOZ2 and XopN_KXO85_ and OsXNP *in planta*. The coding sequences of *xopN*_*KXO85*_, *OsVOZ2*, *OsXNP*, and *OsVOZ1* were cloned into pDEST-SCYNE(R)^GW^ and pDEST-SCYCE(R)^GW^ using the Gateway recombination system to yield pSCYNE(R)-XopN, pSCYCE(R)-OsVOZ2, pSCYCE(R)-OsXNP, and pSCYCE(R)-OsVOZ1, respectively (Table S3). When *Agrobacterium* cells carrying both plasmids were infiltrated into *N* *. benthamiana* leaves, the super cyan fluorescent protein (SCFP3A) signal was detected in the cytoplasm of the *N* *. benthamiana* cells (Figure 2C). As a positive control, we used the Cnx6 and Cnx7 interaction model to form a complex of molybdopterin synthase in *A. thaliana* using pEXP-SCYNE(R)-Cnx7 and pEXP-SCYCE(R)-Cnx6 [40]. These results indicate that XopN_KXO85_ interacts with OsVOZ2 and OsXNP in the cytoplasm of *N* *. benthamiana* cells. However, XopN_KXO85_ does not interact with OsVOZ1 in *N* *. benthamiana* cells (Figure 2C).

### Subcellular localization of XopN_KXO85_, OsVOZ2, and OsXNP

**Figure 3:**
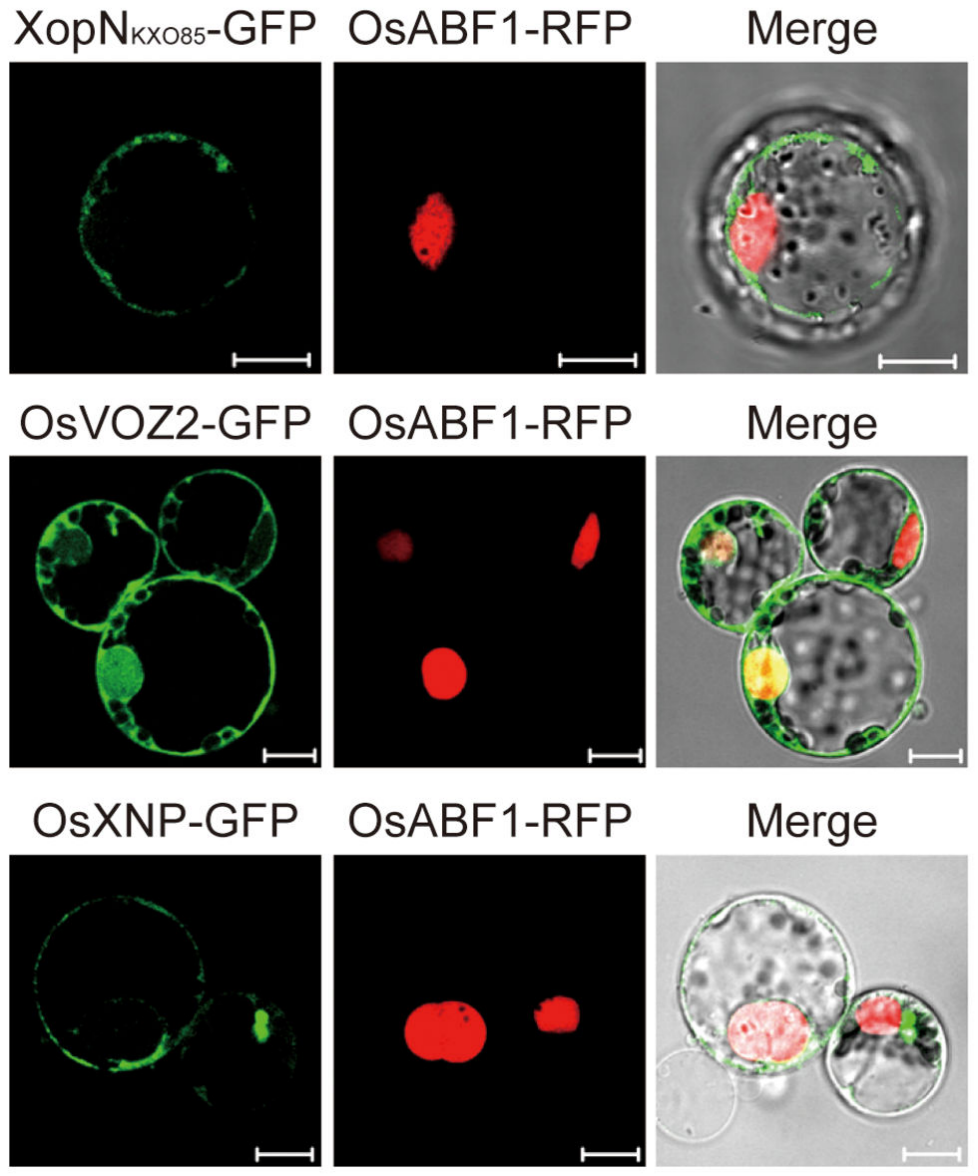
Localization of XopN_KXO85_, OsVOZ2, and OsXNP in plant cells. Subcellular localization of the XopN_KXO85_-GFP, OsVOZ2-GFP, and OsXNP-GFP fusion proteins in maize mesophyll cells. OsABF1-RFP was used as a nuclear marker. GFP (green) fluorescence was merged with RFP (red) fluorescence. Bars = 10 µm.

To determine their subcellular localizations, XopN_KXO85_, OsVOZ2, and OSXNP were tagged with GFP at their C-termini in p2GWF7-XopN, p2GWF7-OsVOZ2, and p2GWF7-OsXNP, respectively (Table S3). In transient expression assays using maize mesophyll protoplasts, GFP signals from XopN-GFP and OsXNP-GFP were mostly detected in the cytoplasm, whereas those from OsVOZ2-GFP were detected in both the cytoplasm and the nucleus compared to the nuclear marker OsABF1-RFP (Figure 3). These data indicate that XopN_KXO85_ and OsXNP are localized in the cytoplasm, whereas OsVOZ2 is localized in a nuclear and cytoplasm (Figure 3).

### Interactions between XopN_KXO85_ and OsVOZ2 are important for *Xoo* virulence in rice

**Figure 4:**
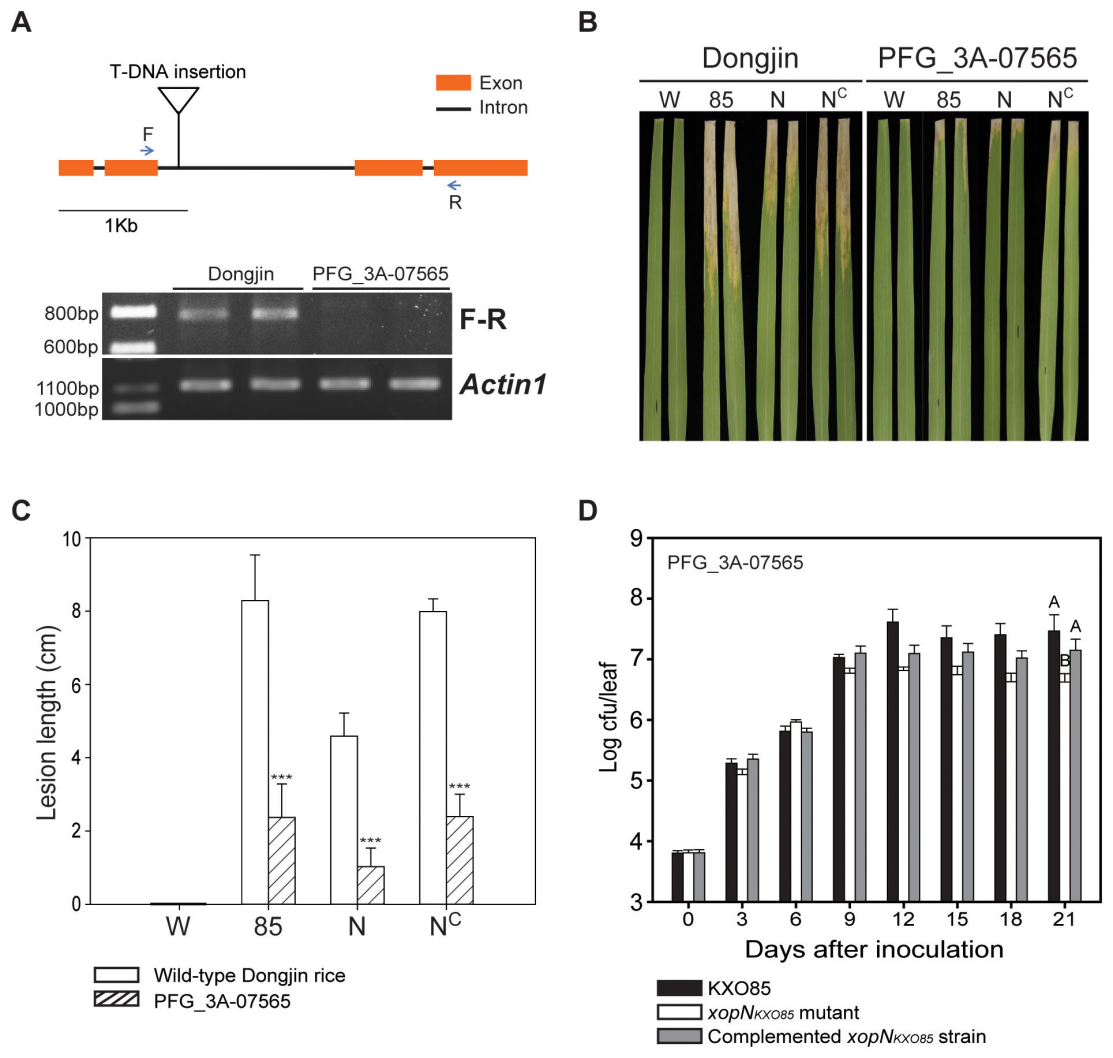
Virulence assay in wild-type Dongjin rice and the OsVOZ2 mutant line PFG_3A-07565. **A**. Schematic representation of the T-DNA insertion in OsVOZ2 T_7_ transgenic rice. *OsVOZ2* consists of four exons (orange boxes) and three introns (line between the orange boxes). The T-DNA was located in the second intron from the translational start site. F and R are the primers used for RT-PCR analysis, which showed the expected size of *OsVOZ2* in wild-type Dongjin but not in the *OsVOZ2* mutant rice PFG_3A-07565. Actin1 was used for normalization of the cDNA quantity. **B**. Virulence assay of the *xopN*_*KXO85*_ mutant in wild-type Dongjin rice and OsVOZ2 mutant rice. W, water; 85, KXO85; N, KXO85 *xopN*_*KXO85*_::EZ-Tn*5*; and N^C^, KXO85 *xopN*_*KXO85*_::EZ-Tn*5* (pML122G2). Photographs were taken 21 days after inoculation. **C**. Measurement of disease severity in flag leaves of wild-type Dongjin rice (□) and OsVOZ2 mutant rice (▨). W, water; 85, KXO85; N, KXO85 *xopN*_*KXO85*_::EZ-Tn*5*; and N^C^, KXO85 *xopN*_*KXO85*_::EZ-Tn*5* (pML122G2). Lesion lengths were determined 21 days after inoculation. Vertical error bars indicate the standard deviation (SD). The statistical significance was determined using a two-way ANOVA as compared to wild-type Dongjin rice with the post hoc Tukey HSD test (***, P<0.001). **D**. Growth patterns of the KXO85, *xopN*_*KXO85*_ mutant, and complemented *xopN*_*KXO85*_ mutant in the flag leaves of OsVOZ2 mutant rice (PFG_3A-07565). The data are the average values of three replicates; vertical bars indicate the error ranges (±SD). The bacterial populations were assessed every 3 days after inoculation. Different letters at day 21 indicate significant differences (P<0.05) as determined by a one-way ANOVA (P<0.001) followed by post hoc Tukey HSD analysis.

To determine whether OsVOZ2 and its interactions with XopN_KXO85_ are critical for *Xoo* virulence, the *OsVOZ2* knockout mutant line PFG_3A-07565 from the rice T-DNA Insertion Sequence Database (http://signal.salk.edu/cgi-bin/RiceGE) [41] was inoculated with wild-type KXO85. In the mutant line PFG_3A-07565, T-DNA is inserted 929 nucleotides downstream from the translational start site of *OsVOZ2*. RT-PCR analysis detected *OsVOZ2* transcript in wild-type Dongjin but not in the *OsVOZ2* mutant line PFG_3A-07565 (Figure 4A), which confirmed knockout mutation in *OsVOZ2*. Wild-type KXO85 and *xopN*_*KXO85*_ mutant strains were inoculated into wild-type Dongjin and the *OsVOZ2* mutant line, and the *xopN*_*KXO85*_ mutant was shown to exhibit reduced virulence in the wild-type Dongjin. However, both strains showed significantly reduced disease severity in the *OsVOZ2* mutant line compared to the wild-type Dongjin (Figure 4B). The *xopN*_*KXO85*_ mutant was less virulent in the *OsVOZ2* mutant line than the wild-type KXO85 (Figure 4B and 4C). The population of *xopN*_*KXO85*_ mutant was smaller than that of wild-type KXO85 in the *OsVOZ2* mutant line (Figure 4D). These results indicate that XopN_KXO85_ is a virulence factor and that its interactions with OsVOZ2 are critical for *Xoo* virulence in rice.

## Discussion

There has been some confusion regarding the roles of Xop of *Xoo* because previous studies have used different *Xoo* strains. The *Xoo* PXO99^A^ strain has 18 non-TAL bacterial effectors [35]. Among these, XopZ_PXO99_ acts as a virulence factor in the *Xoo* PXO99^A^ strain and suppresses plant basal defense mechanisms [35]. XopR_MAFF311018_ was reported as a virulence factor in rice and inhibits the plant basal defense in *A. thaliana* [42]. Nine non-TAL effectors have been identified in the Chinese strain 13751, among which XopR_13751_ has been shown to affect virulence in *Xoo* [43]. In the present study, we chose XopN_KXO85_ to evaluate functional roles in the KXO85 strain and confirmed that it is secreted in an Hrp T3SS-dependent manner, translocated into the plant cytoplasm, and that its gene expression is regulated by HrpX_KXO85_, as reported previously for other *Xoo* strains [13]. Among the Xop homologs in KXO85, we found that XopN_KXO85_ is the most critical for *Xoo* virulence in the Korean strain KXO85. This result is similar to other reports indicating that *xopN*_*Xcv*_ and *xopN*_*Xcc*_ mutants show reduced virulence [26,28].

It is worth noting that different Xop effectors from different *Xoo* strains have been reported to be major Xops involved in *Xoo* virulence. Differences in genetic backgrounds of *Xoo* strains and rice cultivars used for virulence assays may explain why different research groups have reported different Xops as major virulence factors. For example, in one study, a mutation in the *xopN* homolog in *Xoo* PXO99^A^ did not alter disease severity in rice cultivar IR24 that was grown in a growth chamber for 4 weeks [35]. However, it should also be noted that differences in environmental conditions and various rice leaf stages used for inoculation of different *Xoo* strains may also result in different outcomes in virulence assays. In previous studies on *Xoo* PXO99^A^ and Chinese strain 13751, relatively young rice leaves were used for virulence assays in a growth chamber or a greenhouse [35,43], whereas we used flag leaves grown in a paddy field during the regular rice growing season. Disease severity induced by *Xoo* depends on rice leaf stage [36–39]. These observations correspond well with previous reports that the response to *Xoo* in rice depends on the age of the host [36]. Environmental conditions for growing rice and virulence assays are additional factors that may affect disease severity. It will be of interest to determine whether the *xopN*_*PXO99*_ mutant of *Xoo* PXO99^A^ shows differences in virulence assays when the mutant is inoculated into rice flag leaves.

Identification of target proteins of bacterial effectors in their hosts provides a basis for understanding effector functions and their roles in pathogenesis and host defense. XopN_Xcv_ targets a tomato atypical receptor-like kinase1 (TARK1) and four tomato 14-3-3 isoforms (TFT1, TFT3, TFT5, and TFT6) to affect the defense signal mechanism [28]. In *Xoo*, the Xoo1488 of *Xoo* MAFF311018 inhibits OsRLCK185 phosphorylation and the downstream MAPK signaling [31]. Therefore, we postulated that XopN_KXO85_ may interact with known kinases that are involved in signal transduction pathways in rice. However, unlike OsRLCK185 in rice, we found no kinase homologs as XopN_KXO85_ targets but rather two previously unknown rice proteins, OsVOZ2 and OsXNP, were identified based on yeast two-hybrid analysis, pull-down, and BiFC assays.

The AtVOZs were first identified as novel transcription factors in *A. thaliana* [44]. AtVOZs interact with phytochrome B and accelerate flowering time in *A. thaliana* [45]. In the nuclei of *A. thaliana* cells, AtVOZ2 is controlled by light quality in a phytochrome-dependent manner [45]. In addition, AtVOZs are involved in controlling many stress reactions and changing the expression of various stress-related genes, such as those related to drought or freezing responses and pathogens [46]. The genome of the wild-type rice Dongjin has an OsVOZ2 homolog, OsVOZ1, which is also an ortholog of AtVOZ2 and has conserved zinc finger amino acid residues [44]. OsVOZ1 and OsVOZ2 share 60.4% identity (Figure S6). Due to the high degrees of similarity between OsVOZ1 and OsVOZ2, we performed BiFC analysis to determine whether OsVOZ1 is a target protein of XopN_KXO85_. However, there was no evidence of an interaction between XopN_KXO85_ and OsVOZ1 (Figure 2C).

AtVOZ2 interacts with five proteins in *A. thaliana*: phytochrome B (PHY B, At2g18790), guanine nucleotide-binding protein alpha-1 subunit (GP ALPHA1, At2g26300), guanine nucleotide-binding protein subunit beta (AGB1,At4g34460), pirin (PRN, At3g59220), and a hypothetical protein (At4g26410) [45,47]. The most apparent AtVOZ2-dependent phenotype is regulation of flowering period in *A. thaliana* after it interacts with phytochrome B [45]. However, it appears that OsVOZ2 is not involved in determining rice flowering time because we found no noticeable differences in flowering time between wild-type Dongjin and the *OsVOZ2* mutant rice line. Other than our findings indicating that OsVOZ2 is a target of XopN_KXO85_ and is involved in *Xoo* virulence, no other functions have yet been reported in rice.

Another target of XopN_KXO85_ is a putative thiamine synthase, OsXNP, which is present as a single-copy gene in rice. The thiamine synthase gene is related to pathogen-induced defense-responsive protein 8 in Indica rice cultivars. Treatment with thiamine induces callose deposition and hydrogen peroxide accumulation and triggers systemic acquired resistance and transient expression of pathogenesis-related genes against pathogen invasion in rice and several other plants [48,49]. These phenomena are consistent with the observation that thiamine plays important roles in host defense mechanisms against pathogen infection. Therefore, we propose that XopN_KXO85_ interacts with a putative thiamine synthase to hinder thiamine biosynthesis, thereby decreasing the defense of rice against *Xoo* infection. The target proteins of XopN_KXO85_ in rice are completely different from the previously reported targets of XopN_Xcv_. These observations indicate that XopN plays a common role as a virulence factor in *Xcv*, *Xcc*, and *Xoo* but functions in different ways in monocots and dicots, reflecting the different pathogen response mechanisms that arose during the coevolution of pathogens and their hosts.

In addition to roles of XopN_KXO85_ as a virulence factor, OsVOZ2 is also important for *Xoo* virulence because wild-type KXO85 failed to successfully infect OsVOZ2 mutant rice. This suggests that interactions between XopN_KXO85_ and OsVOZ2 in rice increases susceptibility to *Xoo* infection. That is, *Xoo* produces XopN_KXO85_ as an effector molecule and utilizes the host protein OsVOZ2 for successful infection and increased virulence. Although the functions of OsVOZ2 are not fully understood in the interactions between *Xoo* and rice, it is evident that OsVOZ2 is a key factor in *Xoo* virulence in rice.

## Materials and Methods

### Ethics Statement

No specific permits were required for these kinds of field studies. This field is owned by the University Farm, College of Agriculture and life Sciences, Seoul National University. This university farm is located in Suwon, which is approximately 40 kilometers south of the main campus of Seoul National University in Seoul, Republic of Korea. The location is not privately-owned or protected in any way. The field studies did not involve endangered or protected species.

### Bacterial strains

The bacterial strains and plasmids used in this study are listed in Table S3. All of the *Xoo* strains used were derivatives of the parent strain KXO85 (KACC10331). *Escherichia coli* cells were grown at 37°C in Luria-Bertani (LB) broth or on LB agar plates. The *Xoo* strains were grown at 28°C in PS broth (PSB: peptone 1%, sucrose 1%, sodium l-glutamate 0.1%) or PS agar (PSA) plates. Antibiotics were used at the following concentrations: ampicillin, 100 µg/mL; gentamycin, 20 µg/mL; kanamycin, 50 µg/mL; tetracycline, 10 µg/mL; and spectinomycin, 50 µg/mL for *E. coli* strains and cephalexin 10 µg/mL; gentamycin, 10 µg/mL; tetracycline, 2 µg/mL; and kanamycin, 25 µg/mL for *Xoo* strains.

### Transposon insertion and marker-exchange mutagenesis

All recombinant DNA techniques were performed according to standard methods [50]. To generate the *xopN*_*KXO85*_ mutant, the approximately 3-kb *Bam*HI fragment carrying the *xopN*_*KXO85*_ gene from BAC clone G2 (Table S3) of *Xoo* KXO85 was cloned into pML122. EZ-Tn*5*<TET-1> was inserted into the coding region of *xopN*_*KXO85*_ in pML122 by *in vitro* transposition according to the supplier’s instructions (Epicentre) yielding pXopN::EZ-Tn*5* (Table S3). pXopN::EZ-Tn*5* was electroporated into *Xoo* KXO85, and the transformed cells were cultured on PSA medium containing tetracycline. The marker-exchanged mutant *Xoo* KXO85 *xopN*_*KXO85*_::EZ-Tn*5* was isolated and confirmed by Southern hybridization. Transposon insertion and marker-exchange mutagenesis of the other *xop* genes (*xopQ*_*KXO85*_, *xopX*_*KXO85*_, *xopP2*_*KXO85*_, and *xopP1*_*KXO85*_) were performed by the same strategy as described above to generate the *xopN*_*KXO85*_ mutant in *Xoo* KXO85.

### Virulence assay

Rice plants of cultivar Dongjin were grown in a paddy field. The *OsVOZ2* mutant rice seeds (PFG_3A-07565; T_0_ seed) were affirmed by the rice T-DNA Insertion Sequence Database (http://signal.salk.edu/cgi-bin/RiceGE) [41]. The homozygous T_7_ transgenic mutant line of the *OsVOZ2* mutant rice was obtained and confirmed by RT-PCR analysis. Overnight cultures of *Xoo* cells were adjusted to approximately 1.8×10^8^ CFU/mL and inoculated into 3-month-old leaves or fully expanded flag leaves by the scissor clip method [51]. Symptoms were scored by measuring lesion lengths 21 days after inoculation. The growth of *Xoo* cells in plants was determined as described previously [16].

### Quantitative real time RT-PCR analysis

The bacterial strains used were cultured in liquid medium XOM2 [52] or PSB for 24 h. Total RNA was isolated from the wild-type strain KXO85 and KXO85 *hrpX*_*KXO85*_::EZ-Tn*5* using an RNeasy kit (Qiagen) according to the manufacturer’s instructions. A total of 1 µg RNA was reverse transcribed into cDNA using M-MLV reverse transcriptase (Promega) for 1 h at 42°C. RT-PCR products from samples were analyzed on agarose gels and the bacterial 16s rRNA was used as a standard. Quantitative real-time RT-PCR (qRT-PCR) was performed using the cDNA and gene-specific primers (Table S4). The transcription levels were determined by Power SYBR Green PCR Master Mix on an Applied Biosystems 7500 Real-Time PCR System (Applied Biosystems). The thermal cycling parameters were: 95°C for 10 min, followed by 40 cycles of 95°C for 30 s and 60°C for 1 min. Expression of 16S rRNA was used to normalize the expression values in each sample, and relative expression values were determined against the average value of wild-type strain KXO85 using the comparative Ct method.

### Adenylate cyclase assays

To generate the *xopN*-*cya* gene fusion protein, the *xopN*_*KXO85*_ gene was cloned into the *Xba*I and *Xho*I sites of pMLTC to generate pMCXopN (Table S3) followed by transformation into *Xoo* KXO85 and KXO85 *hrpB5*_*KXO85*_::EZ-Tn*5*. For the assay of adenylate cyclase activity in rice leaf tissues, rice leaves were hand-inoculated with bacterial suspension using a needleless syringe. After 12 h, samples were frozen with liquid nitrogen and homogenized in assay buffer supplied with the cAMP Biotrak Enzyme Immunoassay System (GE Healthcare). The level of cAMP in leaf samples was measured by the cAMP Biotrak Enzyme Immunoassay System according to the manufacturer’s directions.

### Yeast two-hybrid assay

A Gal4-based system with Gateway technology (Invitrogen) was used for a yeast two-hybrid assay. The *xopN*_*KXO85*_ gene was amplified by PCR using *Xoo* KXO85 genomic DNA as a template. The PCR primers (Table S5) were flanked with the attB1 and attB2 sites required for the Gateway cloning system. The PCR product was cloned into pDONR222 by BP recombination to generate the entry clone. Subsequently, the *xopN*_*KXO85*_ gene was transferred to the yeast destination bait plasmid pDEST32 by LR recombination resulting in pD32XopN (Table S3). To construct a Dongjin cDNA library, cDNA of approximately 0.5–3 kb was cloned into pDONR222 and subsequently into the prey plasmid pDEST22 by LR recombination yielding pD22Lib (Table S3). pD32XopN contains the DNA-binding domain of Gal4 and the leucine selection marker gene *LEU2*. pD22Lib contains the GAL4 transcription activation domain and the tryptophan selection marker gene *TRP1*. All constructs were checked by restriction enzyme analysis and confirmed by DNA sequencing. pD32XopN (bait) and pD22Lib (prey) were co-transformed into yeast strain MaV203 according to the manufacturer’s protocol (Invitrogen). The transformants were cultured on synthetic complete (SC) medium lacking leucine (–Leu) and tryptophan (–Trp). After 72 h, colonies were picked and mixed with 100 µL of sterile water, and 10 µL of the cell suspension was spotted onto selection plates to screen for expression of the three reporter genes (*HIS3*, *URA3*, and *lacZ*). Growth of the yeast transformants was assessed on SC–Leu–Trp–His supplemented with 0–50 mM 3-amino-1,2,4-triazole (3AT) as a histidine inhibitor and SC–Leu–Trp–Ura. A change in the blue color of the transformants was monitored in the presence of X-Gal (5-bromo-4-chloro-3-indolyl-β-d-galactopyranoside). To check for autoactivation of the reporter genes, pD32XopN, pD22OsVOZ2, and pD22OsXNP were combined with pDEST32 or pDEST22 and tested for autoactivation activity. We used the controls provided by Invitrogen: S (strong control: pEXP ^TM^32/Krev1 + pEXP ^TM^22/RalGDS-wt), W (weak control: pEXP ^TM^32/Krev1 + pEXP ^TM^22/RalGDS-m1), and A (absent control: pEXP ^TM^32/Krev1 + pEXP ^TM^22/RalGDS-m2). Protein expression was confirmed by immunoblotting using anti-GAL4BD (Clontech) and anti-GAL4AD (Clontech) antibodies. Signals were visualized using an Immun-Star WesternC Kit (Bio-Rad).

### *Agrobacterium*-mediated transient expression

*Agrobacterium* infiltration into *N*. *benthamiana* leaves was performed as described previously [28]. Cells of *A. tumefaciens* strain C58C1 (pCH32) [53] were cultured at 28°C for 2 days on LB agar medium containing 50 µg/mL kanamycin and 2.5 µg/mL tetracycline. The recombinant agrobacteria were grown in 10 mL LB liquid medium supplemented with appropriate antibiotics at 28°C and then harvested by centrifugation. The cell pellet was resuspended in buffer (10 mM MES, pH 5.6, 10 mM MgCl_2_, and 150 µM acetosyringone), adjusted to a final OD_600_ of 0.6, and then incubated for 3 h at room temperature before inoculation. Cells were hand-infiltrated onto *N*. *benthamiana* leaves using a needleless 1 mL syringe. Inoculated plants were incubated at 26°C in a growth chamber for 1 to 2 days.

### *In vivo* pull-down assay

To generate plasmids for Ni-NTA affinity pull-down assays, pENTR-XopN, pENTR-OsVOZ2, and pENTR-OsXNP were recombined into pGWB8, pGWB12, and pGWB11 by LR recombination yielding pGWB8-XopN, pGWB12-OsVOZ2, and pGWB11-OsXNP, respectively (Table S3). XopN_KXO85_ was tagged with 6× His, OsVOZ2 was tagged with Flag at the N-terminal, and OsXNP was tagged with Flag at the C-terminal. The bacterial suspensions of *A. tumefaciens* coexpressing XopN_KXO85_-6× His/Flag-OsVOZ2 and XopN_KXO85_-6× His/OsXNP-Flag were hand-infiltrated into *N*. *benthamiana* leaves. At 30 h after infiltration, the leaves were frozen with liquid nitrogen and then macerated in extraction buffer (100 mM sodium phosphate, pH 7.4, 20 mM imidazole, and 0.15% Triton X-100). Homogenized samples were mixed for 1 h at 4°C and centrifuged for 15 min at 17000 × *g* at 4°C. The soluble extracts were incubated with 30 µL 50% slurry of Ni-NTA Superflow Agarose (Qiagen). Ni-NTA agarose was retrieved by centrifugation and washed three times with extraction buffer, and proteins were eluted with 8 M urea sample buffer followed by Western blotting analysis using anti-6× His (Qiagen) and anti-FLAG (Sigma) antibodies. Signals were visualized using detection solution (Immun-Star WesternC Kit; Bio-Rad).

### BiFC

The coding regions of *xopN*_*KXO85*_, *OsVOZ2*, *OsXNP*, and *OsVOZ1* were amplified by PCR using proofreading DNA polymerase and appropriate primers (Table S6) and cloned into the Gateway entry vector pENTR D TOPO (Invitrogen) yielding pENTR-XopN, pENTR-OsVOZ2, pENTR-OsXNP, and pENTR-OsVOZ1, respectively. pENTR-XopN, pENTR-OsVOZ2, pENTR-OsXNP, and pENTR-OsVOZ1 were recombined into the Gateway binary BiFC vectors pDEST-SCYNE(R)^GW^ and pDEST-SCYCE(R)^GW^ using LR recombinase according to the manufacturer’s instructions (Invitrogen) yielding pSCYNE-XopN, pSCYCE-OsVOZ2, pSCYCE-OsXNP, and pSCYCE-OsVOZ1, respectively (Table S3). The constructs were confirmed by DNA sequencing and transformed into *A. tumefaciens* C58C1 (pCH32) for transient expression in *N* *. benthamiana* as described above. SCFP signals were detected using a confocal laser scanning microscope (Leica Microsystems) 26 h after infiltration.

### Localization of OsVOZ2, OsXNP, and XopN_KXO85_

The *OsVOZ2*, *OsXNP*, and *xopN*_*KXO85*_ genes in pENTR D TOPO were cloned into the destination vector p2GWF7 to create a C-terminal GFP fusion [54] using the Gateway LR recombinase (Invitrogen). The constructs were introduced into maize mesophyll protoplasts by polyethylene glycol–calcium-mediated transformation [55,56]. The protoplasts were examined after incubation for 12–24 h. OsABF1-RFP was used as a nuclear marker [57].

### Microscopy

We used confocal laser scanning microscopes (SCFP: TCS SP5; Leica Microsystems; GFP: LSM510 META; Carl Zeiss) to detect the SCFP and GFP signals. The excitation and emission wavelengths were SCFP (458 nm and 465–480 nm, respectively) and GFP (488 nm and 500–525 nm, respectively).

### Statistical analysis

JMP^®^ 10 software (SAS Institute) was used for statistical analysis. Statistical significance was determined by a one-way or a two-way ANOVA with Tukey HSD post-test.

## Supporting Information

Figure S1 **Genetic organization of five *xop* genes and EZ-Tn*5* insertion positions in the *Xoo* KXO85 genome.** The vertical bar with black open triangle indicates the position of the EZ-Tn*5* insertion. Arabic numerals on the left and right sides indicate the base position in the *Xoo* KXO85 genome.
(DOC)

Figure S2 ***XopN*_*KXO85*_ expression is regulated by HrpX_KXO85_ in *Xoo* KXO85.** **A**. Expression profiles of *XopN*_*KXO85*_ regulated by HrpX_KXO85_ based on RT-PCR (left panel) and qRT-PCR (right panel) analyses. The 16S rRNA gene of KXO85 was used for normalization of the cDNA quantity and expression value. WT, *Xoo* KXO85; X, *Xoo* KXO85 *hrpX*_*KXO85*_::EZ-Tn5; PSB, bacterial strains were incubated in PSB (1% peptone, 1% sucrose, and 0.1% sodium l-glutamate); XOM2, bacterial strains were incubated in *hrp-*inducing medium XOM2. Vertical error bars indicate the standard deviation. **B**. The PIP box (TTCGG-N_15_-TTCTG) is located near *XopN*_*KXO85*_ in the KXO85 genome.
(DOC)

Figure S3 **Genetic map of the *hrpB5*_*KXO85*_ mutant and cAMP measurement in rice leaves.** **A**. The vertical bar with a black open triangle indicates the position of the EZ-Tn*5* insertion in *hrpB5*_*KXO85*_ in the KXO85 genome. The numbers on the left and right sides indicate the base positions in the KXO85 genome. **B**. Levels of cAMP in rice leaves. WT, KXO85; B5, KXO85 *hrpB5*_*KXO85*_::EZ-Tn5; N, KXO85 (pMC*xopN*); B5-N, KXO85 *hrpB5*_*KXO85*_::EZ-Tn*5* (pMC*xopN*); TC, KXO85 (pMLTC)-vector control; B5-TC, KXO85 *hrpB5*_*KXO85*_::EZ-Tn*5* (pMLTC)-vector control; and W, water. For the cAMP assays, each data point represents the average of three replicate samples with error bars indicating the standard deviation.
(DOC)

Figure S4 **Self-activation test and Western blot analysis showing expression of the yeast plasmid constructs in yeast two-hybrid screening.** **A**. Self-activation tests were conducted using pD32XopN + pDEST22, pDEST32 + pD22OsVOZ2, pDEST32 + pD22OsXNP, and pDEST32 + pDEST22. **B**. Total proteins were extracted from the indicated yeast strains. Anti-GAL4BD and anti-GAL4AD antibodies were used for immunoblotting. M, size marker; 1, pD32XopN and pD22OsVOZ2; 2, pD32XopN and pD22OsXNP; and 3, pDEST32 and pDEST22. The expected molecular weights of the proteins were as follows: GAL4BD-XopN_KXO85_ = 94. 4 kDa; GAL4AD-OsVOZ2 = 84.8 kDa; GAL4AD-OsXNP = 53.2 kDa; GAL4BD: 18. 4 kDa; and GAL4AD: 14.9 kDa. The arrow (◀) indicates the position of expressed protein.
(DOC)

Figure S5 **The amino acid sequence alignment of *A. thaliana* VOZ2 and OsVOZ2 using the ClustalW2 multiple alignment program.**
(DOC)

Figure S6 **The amino acid sequence alignment of OsVOZ1 and OsVOZ2 using the ClustalW2 multiple alignment program. The red box represents conserved residues possibly forming a functional zinc-coordinating motif.**
(DOC)

Table S1 **Characteristics of five predicted *xop* genes from *Xoo* KXO85.**
(DOC)

Table S2 **Identification of OsVOZ2 and OsXNP.**
(DOC)

Table S3 **Bacterial strains and plasmids.**
(DOC)

Table S4 **Primers used for qRT–PCR of *xopN*_KXO85_.**
(DOC)

Table S5 **Primers used for yeast two-hybrid system.**
(DOC)

Table S6 **Primers used for BiFC, localization, and *in vivo* pull-down assay.**
(DOC)
