# Supplementary figures and images for: Xanthomonas oryzae pv. oryzae Type III Effector XopN Targets OsVOZ2 and a Putative Thiamine Synthase as a Virulence Factor in Rice
Source: PLoS One. 2013 Sep 3;8(9):e73346. doi: 10.1371/journal.pone.0073346 (PMC3760903; doi:10.1371/journal.pone.0073346)

**Figure S1**


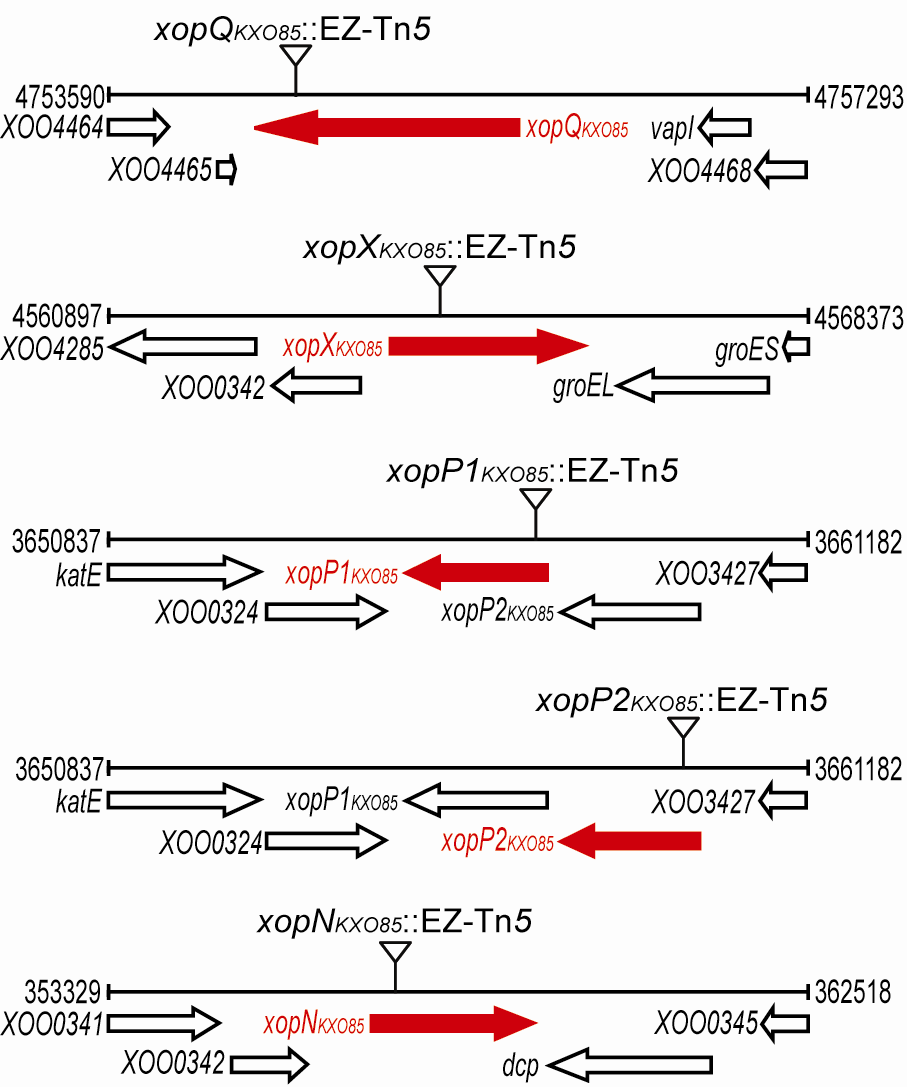

Supplement: Figure S1 — Genetic organization of five xop genes and EZ-Tn5 insertion positions in the Xoo KXO85 genome. The vertical bar with black open triangle indicates the position of the EZ-Tn5 insertion. Arabic numerals on the left and right sides indicate the base position in the Xoo KXO85 genome. (DOC) [file pone.0073346.s001.doc]

**Figure S2**


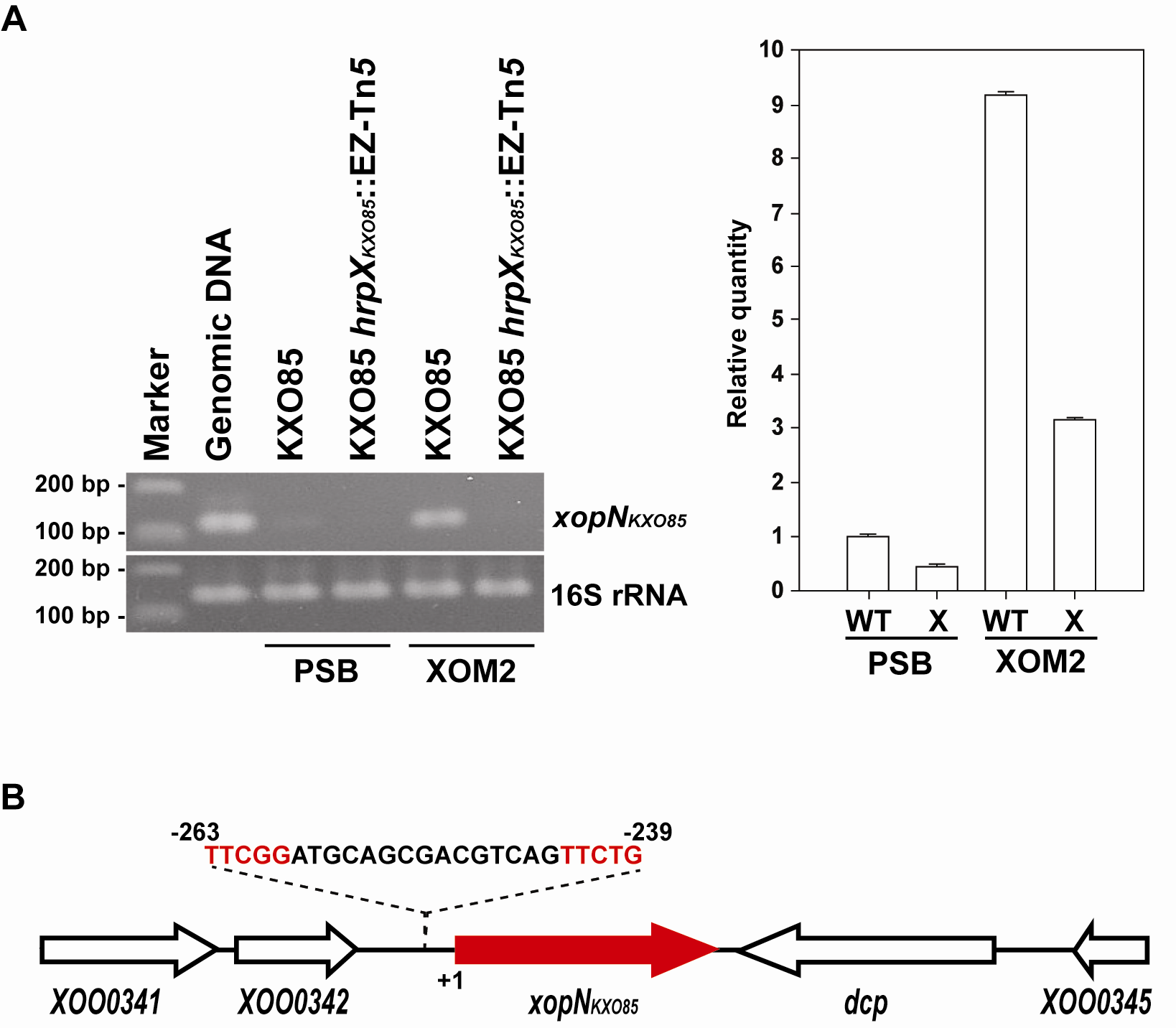

Supplement: Figure S2 — XopNKXO85 expression is regulated by HrpXKXO85 in Xoo KXO85. A. Expression profiles of XopN KXO85 regulated by HrpXKXO85 based on RT-PCR (left panel) and qRT-PCR (right panel) analyses. The 16S rRNA gene of KXO85 was used for normalization of the cDNA quantity and expression value. WT, Xoo KXO85; X, Xoo KXO85 hrpX KXO85::EZ-Tn5; PSB, bacterial strains were incubated in PSB (1% peptone, 1% sucrose, and 0.1% sodium l-glutamate); XOM2, bacterial strains were incubated in hrp-inducing medium XOM2. Vertical error bars indicate the standard deviation. B. The PIP box (TTCGG-N15-TTCTG) is located near XopN KXO85 in the KXO85 genome. (DOC) [file pone.0073346.s002.doc]

**Figure S3**


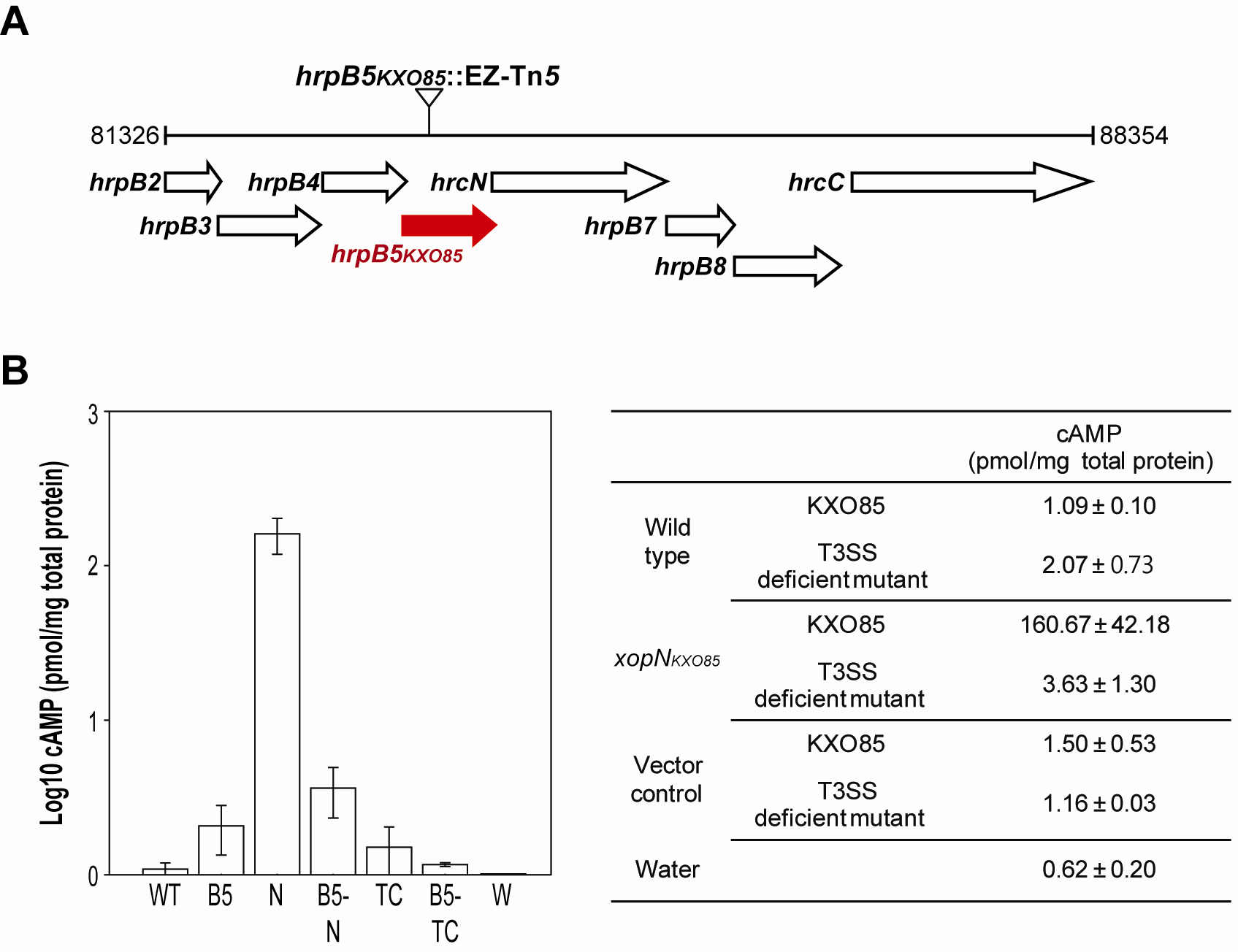

Supplement: Figure S3 — Genetic map of the hrpB5KXO85 mutant and cAMP measurement in rice leaves. A. The vertical bar with a black open triangle indicates the position of the EZ-Tn5 insertion in hrpB5 KXO85 in the KXO85 genome. The numbers on the left and right sides indicate the base positions in the KXO85 genome. B. Levels of cAMP in rice leaves. WT, KXO85; B5, KXO85 hrpB5 KXO85::EZ-Tn5; N, KXO85 (pMCxopN); B5-N, KXO85 hrpB5 KXO85::EZ-Tn5 (pMCxopN); TC, KXO85 (pMLTC)-vector control; B5-TC, KXO85 hrpB5 KXO85::EZ-Tn5 (pMLTC)-vector control; and W, water. For the cAMP assays, each data point represents the average of three replicate samples with error bars indicating the standard deviation. (DOC) [file pone.0073346.s003.doc]

**Figure S4**

**
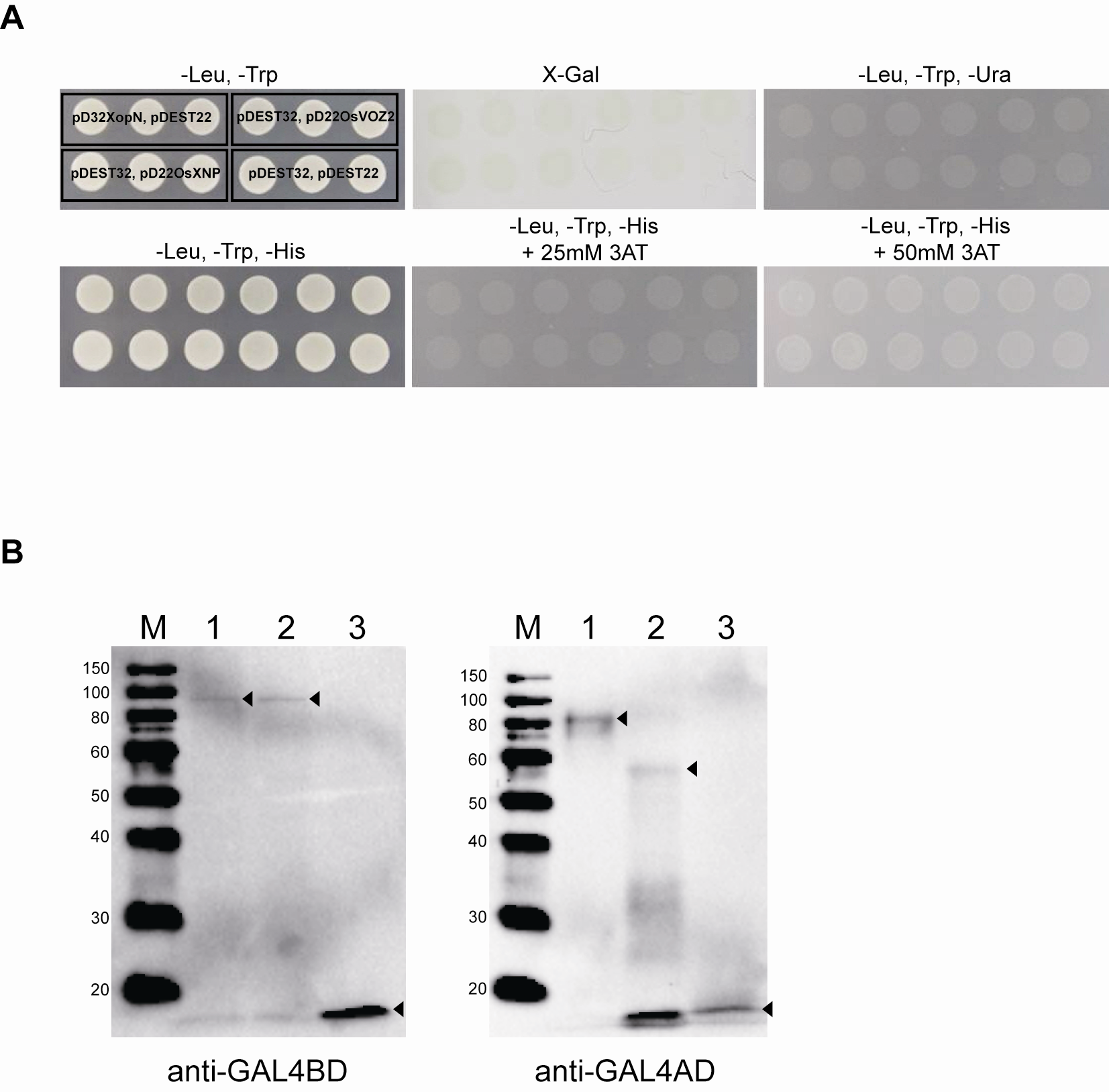
**

Supplement: Figure S4 — Self-activation test and Western blot analysis showing expression of the yeast plasmid constructs in yeast two-hybrid screening. A. Self-activation tests were conducted using pD32XopN + pDEST22, pDEST32 + pD22OsVOZ2, pDEST32 + pD22OsXNP, and pDEST32 + pDEST22. B. Total proteins were extracted from the indicated yeast strains. Anti-GAL4BD and anti-GAL4AD antibodies were used for immunoblotting. M, size marker; 1, pD32XopN and pD22OsVOZ2; 2, pD32XopN and pD22OsXNP; and 3, pDEST32 and pDEST22. The expected molecular weights of the proteins were as follows: GAL4BD-XopNKXO85 = 94. 4 kDa; GAL4AD-OsVOZ2 = 84.8 kDa; GAL4AD-OsXNP = 53.2 kDa; GAL4BD: 18. 4 kDa; and GAL4AD: 14.9 kDa. The arrow (◀) indicates the position of expressed protein. (DOC) [file pone.0073346.s004.doc]

**Figure S5**


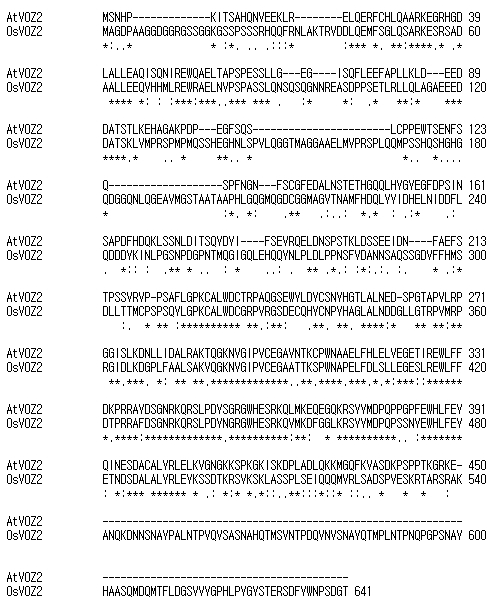

Supplement: Figure S5 — The amino acid sequence alignment of A. thaliana VOZ2 and OsVOZ2 using the ClustalW2 multiple alignment program. (DOC) [file pone.0073346.s005.doc]

**Figure S6**


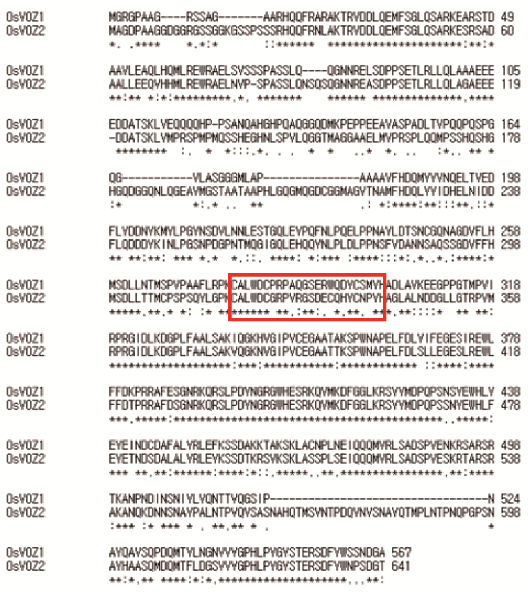

Supplement: Figure S6 — The amino acid sequence alignment of OsVOZ1 and OsVOZ2 using the ClustalW2 multiple alignment program. The red box represents conserved residues possibly forming a functional zinc-coordinating motif. (DOC) [file pone.0073346.s006.doc]
