# Supplementary material for: Xanthomonas oryzae pv. oryzae Type III Effector XopN Targets OsVOZ2 and a Putative Thiamine Synthase as a Virulence Factor in Rice
Source: PLoS One. 2013 Sep 3;8(9):e73346. doi: 10.1371/journal.pone.0073346 (PMC3760903; doi:10.1371/journal.pone.0073346)
Supplement: Table S1 — Characteristics of five predicted xop genes from Xoo KXO85. (DOC) [file pone.0073346.s007.doc]

**Table S1 Characteristics of five predicted *xop* genes from *Xoo* KXO85.**

| Gene | Locus in *X o o* KXO85 | ORF size (bp) | a. a | M. W (Dalton) | pI | Conserved Domain Search  by NCBI database | Homolog |
| --- | --- | --- | --- | --- | --- | --- | --- |
| *xopQ* | XOO4466 | 1395 | 464 | 50022.43 | 6.975 | Nucleoside hydrolase (4) | HopQ1  (*P. syringae* pv. *phaseolicola*) |
| *xopX* | XOO4287 | 2109 | 702 | 74081.64 | 6.415 | - | Hypothetical protein  (*X. campestris* pv. *vesicatoria*) |
| *xopP1* | XOO3425 | 2133 | 710 | 77646.56 | 8.502 | - | Hypothetical protein  (*X. campestris* pv. *vesicatoria*) |
| *xopP2* | XOO3426 | 2142 | 713 | 77932.93 | 8.636 | - | Hypothetical protein  (*X. campestris* pv. *vesicatoria*) |
| *xopN* | XOO0343 | 2163 | 720 | 76020.12 | 8.445 | - | HopAU1  (*P. syringae* pv. *phaseolicola*) |
