# Supplementary material for: Xanthomonas oryzae pv. oryzae Type III Effector XopN Targets OsVOZ2 and a Putative Thiamine Synthase as a Virulence Factor in Rice
Source: PLoS One. 2013 Sep 3;8(9):e73346. doi: 10.1371/journal.pone.0073346 (PMC3760903; doi:10.1371/journal.pone.0073346)
Supplement: Table S2 — Identification of OsVOZ2 and OsXNP. (DOC) [file pone.0073346.s008.doc]

**Table S2** **Identification of OsVOZ**2 and OsXNP.

| Gene | Accession No. | Gene ID | Gene product | Homolog  (GenBank Accession No.) |
| --- | --- | --- | --- | --- |
| *OsVOZ2* | NP_001056041.1 | Os05g0515700  [*Oryza sativa* Japonica Group] | Hypothetical protein | VOZ2  (AEC10117.1) |
| *OsXNP* | NP_001059841 | Os07g0529600  [*Oryza sativa* Japonica Group] | Putative thiamine biosynthesis protein | thiamine biosynthetic enzyme  (AFO59577.1) |
