# Supplementary material for: Xanthomonas oryzae pv. oryzae Type III Effector XopN Targets OsVOZ2 and a Putative Thiamine Synthase as a Virulence Factor in Rice
Source: PLoS One. 2013 Sep 3;8(9):e73346. doi: 10.1371/journal.pone.0073346 (PMC3760903; doi:10.1371/journal.pone.0073346)
Supplement: Table S3 — Bacterial strains and plasmids. (DOC) [file pone.0073346.s009.doc]

**Table S3** **Bacterial strains and plasmids.**

| **Strain or plasmid** | **Characteristics^a)^** | **Reference or source** |
| --- | --- | --- |
| ***Xanthomonas oryzae* pv. *oryzae*** |  |  |
| KXO85 | KACC10331, wild-type, Korean race 1 strain that is virulent to rice carrying the *Xa21* resistance gene, Ceph^R^ | [1] |
| KXO85 *xopQ_KXO85_*::EZ-Tn*5* | *xopQ* mutant of KXO85, Tet^R^ | This study |
| KXO85 *xopX_KXO85_*::EZ-Tn*5* | *xopX* mutant of KXO85, Tet^R^ | This study |
| KXO85 *xopP1_KXO85_*::EZ-Tn*5* | *xopP1* mutant of KXO85, Tet^R^ | This study |
| KXO85 *xopP1_KXO85_*::EZ-Tn*5* (pML122B7) | *xopP1* mutant complemented with pML122G2, Tet^R^, Gm^R^ | This study |
| KXO85 *xopP2_KXO85_*::EZ-Tn*5* | *xopP2* mutant of KXO85, Tet^R^ | This study |
| KXO85 *xopP2_KXO85_*::EZ-Tn*5* (pML122B7) | *xopP2* mutant complemented with pML122G2, Tet^R^, Gm^R^ | This study |
| KXO85 *xopN_KXO85_*::EZ-Tn*5* | *xopN* mutant of KXO85, Tet^R^ | This study |
| KXO85 *xopN_KXO85_*::EZ-Tn*5* (pML122G2) | *xopN* mutant complemented with pML122G2, Tet^R^, Gm^R^ | This study |
| KXO85 *hrpX_KXO85_*::EZ-Tn*5* | *hrpX* mutant of KXO85, Km^R^ | This study |
| KXO85 *hrpB5_KXO85_*::EZ-Tn*5* | Type III secretion system-deficient mutant of KXO85, Km^R^ | This study |
| ***Escherichia coli*** |  |  |
| DH5α | F^-^ Φ80d*lacZ*Δ*M15* Δ(*lacZYA-argF*)*U169 endA1 recA1 hsdR17* (r_K_^-^m_K_^+^) *deoR thi-1 supE44 λ-gyrA96 relA1* | Gibco BRL |
| DB3.1 | F^-^ *gyrA462* *endA1* Δ(*sr1-recA*) *mcrB* *mrr* *hsdS20*(*rB-, Mb-*) *supE44* *ara-14* *galK2* *lacY1* *proA2* *rpsL20*(Sp^R^) *xyl-5 λ- leu mtl1* | Invitrogen |
| **BAC clone** |  |  |
| G2 | BAC plasmid containing XOO0279 to XOO0382 ORFs in *Xoo* KXO85 genome | [1] |
|  |  |  |
|  |  |  |
| (Continued) |  |  |
| ***Saccharomyces cerevisiae*** |  |  |
| MaV203 | MATα, *leu*2–3,112, *trp*1–901, *his*3Δ200, *ade*2–101, *gal*4Δ, *gal*80Δ, *SPAL*10::*URA*3, *GAL*1::*lac*Z, *HIS*3_UAS GAL1_::*HIS*3@*LYS*2, *can*1^R^, *cyh*2^R^ | Invitrogen |
| ***Agrobacterium tumefaciens*** |  |  |
| C58C1 (pCH32) | Rif^R^, Tet^R^ | [2] |
| **Plasmids** |  |  |
| pML122 | OriV, OriT, Gm^R^, *p*Nm (*npt*II), broad host range expression vector | [3] |
| pXopN::EZ-Tn*5* | EZ-Tn*5*<TET-1> was inserted into the coding region of *xopN_KXO85_* in pML122 | This study |
| pML122G2 | pML122 containing the *xopN_KXO85_* coding sequence and flanking region cloned into *Bam*HI site | This study |
| pML122B7 | pML122 containing the *xopP2_KXO85_* and *xopP1_KXO85_* coding sequence and flanking region cloned into *Hin*dIII site | This study |
| pMLTC | Cya reporter gene was cloned into pML122, Gm^R^ | This study |
| pMCXopN | pMLTC containing the *xopN_KXO85_* coding sequence, Gm^R^ | This study |
| pDONR222 | Gateway pDONR vector with pUC origin, T7 promoter/priming site, M13 forward (-20) and reverse priming sites; *rrnB* T1 and T2 transcription terminators, *att*P1 and *att*P2 sites, *ccd*B gene, Km^R^, Cm^R^ | Invitrogen |
| pDONR-XopN | pDONR222 containing the *xopN_KXO85_* gene | This study |
| pDEST32 | Gateway destination bait vector, GAL4 DNA binding domain (GAL4 DBD), *ccd*B gene, *LEU*2 gene, Cm^R^, Gm^R^ | Invitrogen |
| pDEST22 | Gateway destination prey vector, GAL4 activation domain (GAL4 AD), *ccd*B gene, *TRP1* gene, Cm^R^, Amp^R^ | Invitrogen |
| pD32xopN | pDEST32 containing *xopN_KXO85_* coding sequence. Gm^R^ | This study |
| pD22Lib | pDEST22 containing the approximately 0.5–3 kb fragment of rice cDNA Library, Amp^R^ | This study |
| pD22OsVOZ2 | pDEST22 containing the *OsVOZ2* sequence of rice, Amp^R^ | This study |
| pD22OsXNP | pDEST22 containing the *OsXNP* sequence of rice, Amp^R^ | This study |
|  |  |  |
| (Continued) |  |  |
| pENTR D TOPO | *att*L1 and *att*L2 sites for site-specific recombination of the entry clone with a gateway destination vector, Km^R^ | Invitrogen |
| pENTR-XopN | pENTR D TOPO harboring the *xopN_KXO85_* (without the stop codon) gene, Km^R^ | This study |
| pENTR-OsVOZ2 | pENTR D TOPO harboring the *OsVOZ2* (without the stop codon) gene, Km^R^ | This study |
| pENTR-OsXNP | pENTR D TOPO harboring the *OsXNP* (without the stop codon) gene, Km^R^ | This study |
| pENTR-OsVOZ1 | pENTR D TOPO harboring the *OsVOZ1* (without the stop codon) gene, Km^R^ | This study |
| pDEST-SCYNE(R)^GW^ | The binary BiFC gateway destination vector, SCFP3A N-terminus, Flag linker, Km^R^, Cm^R^ | [4] |
| pDEST-SCYCE(R)^GW^ | Binary BiFC gateway destination vector, SCFP3A C-terminus, HA linker, Km^R^, Cm^R^ | [4] |
| pEXP-SCYNE(R)-Cnx7 | *A*. *thaliana* gene Cnx7 was cloned into pDEST-SCYNE(R)^GW^ using gateway LR recombination. | [4] |
| pEXP-SCYCE(R)-Cnx6 | *A*. *thaliana* gene Cnx6 was cloned into pDEST-SCYCE(R)^GW^ using gateway LR recombination. | [4] |
| pSCYNE(R)-XopN | pENTR-XopN was recombined with pDEST-SCYNE(R)^GW^ using gateway LR recombination. | This study |
| pSCYCE(R)-OsVOZ2 | pENTR-OsVOZ2 was recombined pDEST-SCYCE(R)^GW^ using gateway LR recombination. | This study |
| pSCYCE(R)-OsXNP | pENTR-OsXNP was recombined pDEST-SCYCE(R)^GW^ using gateway LR recombination. | This study |
| pSCYCE(R)-OsVOZ1 | pENTR-OsVOZ1 was recombined pDEST-SCYCE(R)^GW^ using gateway LR recombination. | This study |

^a)^ Ceph^R^, cephalexin resistance; Gm^R^, gentamycin resistance; Km^R^, kanamycin resistance; Cm^R^, chloramphenicol resistance;

| (Contined) |  |  |
| --- | --- | --- |
| p2GWF7 | C-terminal GFP fusion, *att*R1 and *att*R2 sites for site-specific recombination of the destination clone with a gateway entry vector, Amp^R^ | [5] |
| p2GWF7-XopN | pENTR-XopN was recombined with p2GWF7 using gateway LR recombination. | This study |
| p2GWF7-OsVOZ2 | pENTR-OsVOZ2 was recombined p2GWF7 using gateway LR recombination. | This study |
| p2GWF7-OsXNP | pENTR-OsXNP was recombined p2GWF7 using gateway LR  recombination. | This study |
| pGWB8 | Binary gateway vector, 35S promoter, C-terminal 6xHis epitopes tag, *ccd*B gene, Km^R^, Cm^R^ | [6] |
| pGWB8-XopN | pENTR-XopN was recombined with pGWB8 using gateway LR recombination, Km^R^ | This study |
| pGWB11 | Binary gateway vector, 35S promoter, C-terminal FLAG epitope tag, *ccd*B gene, Km^R^, Cm^R^ | [6] |
| pGWB11-OsXNP | pENTR-OsXNP was recombined pGWB11 using gateway LR recombination, Km^R^ | This study |
| pGWB12 | Binary gateway vector, 35S promoter, N-terminal FLAG epitope tag, *ccd*B gene, Km^R^, Cm^R^ | [6] |
| pGWB12-OsVOZ2 | pENTR-OsVOZ2 was recombined pGWB12 using gateway LR recombination, Km^R^ | This study |

Sp^R^, spectinomycin resistance; Tet^R^, tetracycline resistance; Amp^R^, ampicillin resistance; Nm, neomycin

[1] Lee B-M, Park Y-J, Park D-S, Kang H-W, Kim J-G, et al. (2005) The genome sequence of *Xanthomonas oryzae* pathovar *oryzae* KACC10331, the bacterial blight pathogen of rice. Nucleic Acids Res 33: 577-586.

[2] [Mudgett MB](http://www.ncbi.nlm.nih.gov/pubmed?term=Mudgett MB%5BAuthor%5D&cauthor=true&cauthor_uid=11078519), [Chesnokova O](http://www.ncbi.nlm.nih.gov/pubmed?term=Chesnokova O%5BAuthor%5D&cauthor=true&cauthor_uid=11078519), [Dahlbeck D](http://www.ncbi.nlm.nih.gov/pubmed?term=Dahlbeck D%5BAuthor%5D&cauthor=true&cauthor_uid=11078519), [Clark ET](http://www.ncbi.nlm.nih.gov/pubmed?term=Clark ET%5BAuthor%5D&cauthor=true&cauthor_uid=11078519), [Rossier O](http://www.ncbi.nlm.nih.gov/pubmed?term=Rossier O%5BAuthor%5D&cauthor=true&cauthor_uid=11078519), et al. (2000) Molecular signals required for type III secretion and translocation of the *Xanthomonas campestris* AvrBs2 protein to pepper plants. Proc Natl Acad Sci USA 97: 13324-13329.

[3] Labes M, Pühler A, Simon R (1990) A new family of RSF1010-derived expression and *lac*-fusion broad-host-range vectors for Gram- negative bacteria. Gene 89: 37-46.

[4] Gehl C, Waadt R, Kudla J, Mendel R -R, Hänsch R (2009) New gateway vectors for high throughput analyses of protein-protein interactions by bimolecular fluorescence complementation. Mol Plant 2: 1051-1058.

[5] Karimi M, Inze D, Depicker A (2002) Gateway vectors for *Agrobacterium*-mediated plant transformation. Trends Plant Sci 7: 193-195.

[6] [Nakagawa T](http://www.ncbi.nlm.nih.gov/pubmed?term=Nakagawa T%5BAuthor%5D&cauthor=true&cauthor_uid=17697981), [Kurose T](http://www.ncbi.nlm.nih.gov/pubmed?term=Kurose T%5BAuthor%5D&cauthor=true&cauthor_uid=17697981), [Hino T](http://www.ncbi.nlm.nih.gov/pubmed?term=Hino T%5BAuthor%5D&cauthor=true&cauthor_uid=17697981), [Tanaka K](http://www.ncbi.nlm.nih.gov/pubmed?term=Tanaka K%5BAuthor%5D&cauthor=true&cauthor_uid=17697981), [Kawamukai M](http://www.ncbi.nlm.nih.gov/pubmed?term=Kawamukai M%5BAuthor%5D&cauthor=true&cauthor_uid=17697981), et al. (2007) Development of series of gateway binary vectors, pGWBs, for realizing efficient construction of fusion genes for plant transformation. [J Biosci Bioeng](http://www.ncbi.nlm.nih.gov/pubmed/17697981) 104: 34-41.
