# Supplementary material for: Xanthomonas oryzae pv. oryzae Type III Effector XopN Targets OsVOZ2 and a Putative Thiamine Synthase as a Virulence Factor in Rice
Source: PLoS One. 2013 Sep 3;8(9):e73346. doi: 10.1371/journal.pone.0073346 (PMC3760903; doi:10.1371/journal.pone.0073346)
Supplement: Table S4 — Primers used for qRT–PCR of xopNKXO85. (DOC) [file pone.0073346.s010.doc]

**Table S4 Primers used for qRT–PCR of *xopN_KXO85_*.**

| Primer name^a^ | Sequence (5′→3′) |
| --- | --- |
| XopN cDNA region | CACGGACAATACGCCCAGTTC |
| XopN (RT)-F | ATGAAACCTGCTGCATCCGC |
| XopN (RT)-R | CTCGGACTGTGCAGGCGAAT |
| 16S rRNA cDNA region | TAAGTGAAGAGTTTGATCCTG |
| 16S rRNA (RT)-F | GGCAGGCCTAACACATGCAA |
| 16S rRNA (RT)-R | GTATTAGCGTAAGTTTCCCTAC |

^a^ F, forward primer; R, reverse primer.
