# Supplementary material for: Xanthomonas oryzae pv. oryzae Type III Effector XopN Targets OsVOZ2 and a Putative Thiamine Synthase as a Virulence Factor in Rice
Source: PLoS One. 2013 Sep 3;8(9):e73346. doi: 10.1371/journal.pone.0073346 (PMC3760903; doi:10.1371/journal.pone.0073346)
Supplement: Table S5 — Primers used for yeast two-hybrid system. (DOC) [file pone.0073346.s011.doc]

**Table S5** **Primers used for yeast two-hybrid system.**

| Primer name^a^ | Sequence (5′→3′) |
| --- | --- |
| attB1xopN-F | GGGGACAAGTTTGTACAAAAAAGCAGGCTTGATGAAACCTGCTGCATCCGCC |
| attB2xopN-R | GGGGACCACTTTGTACAAGAAAGCTGGGTCTATTACGCCGGCAGTGCCCG |
| Bait-F | AACCGAAGTGCGCCAAGTGTCTG |
| Bait and Prey-R | AGCCGACAACCTTGATTGGAGAC |
| Prey-F | TATAACGCGTTTGGAATCACT |

^a^ F, forward primer; R, reverse primer.
