# Supplementary material for: Xanthomonas oryzae pv. oryzae Type III Effector XopN Targets OsVOZ2 and a Putative Thiamine Synthase as a Virulence Factor in Rice
Source: PLoS One. 2013 Sep 3;8(9):e73346. doi: 10.1371/journal.pone.0073346 (PMC3760903; doi:10.1371/journal.pone.0073346)
Supplement: Table S6 — Primers used for BiFC, localization, and in vivo pull-down assay. (DOC) [file pone.0073346.s012.doc]

**Table S6** **Primers used for BiFC, localization, and *in vivo* pull-down assay.**

| Primer name^a^ | Sequence (5′→3′) |
| --- | --- |
| XopN (D-TOPO)-F | CACCATGAAACCTGCTGCATC |
| XopN (D-TOPO)-R | CGCCGGCGGCAGTGCCCGAT |
| OsVOZ2 (D-TOPO)-F | CACCATGGCCGGCGATCCG |
| OsVOZ2 (D-TOPO)-R | TGTCCCGTCACTAGGGTTC |
| OsXNP (D-TOPO)-F | CACCATGGCAGCCATGGCCA |
| OsXNP (D-TOPO)-R | GGCGTCCACGATCTCGCCGT |
| OsVOZ1 (D-TOPO)-F | CACCATGGGGAGGGGCCCG |
| OsVOZ1 (D-TOPO)-R | AGCTCCATCATTTGAGCTCC |

^a^ F, forward primer; R, reverse primer.
